# Epidemiological study of pediatric trauma in a reference hospital in Curitiba

**DOI:** 10.1590/0100-6991e-20233447-en

**Published:** 2023-07-26

**Authors:** FERNANDA GLUS SCHARNOSKI, ISADORA MEZZOMO DESCONSI, MARC DOMIT WERNER LINNENKAMP, HECTOR SBARAINI FONTES, CAROLINE DE OLIVEIRA PEREIRA, RODRIGO KRIEGER MARTINS, LUCAS MANSANO SARQUIS

**Affiliations:** 1 - Universidade Positivo, Faculdade de Medicina - Curitiba - PR - Brasil; 2 - Pontifícia Universidade Católica do Paraná, Faculdade de Medicina - Curitiba - PR - Brasil; 3 - Hospital do Trabalhador, Serviço de Cirurgia Geral - Curitiba - PR - Brasil

**Keywords:** Epidemiology, Traumatology, Child, Adolescent, Wounds and Injuries, Epidemiologia, Traumatologia, Criança, Adolescente, Ferimentos e Lesões

## Abstract

**Objective:**

to analyze the prevalence of types of trauma, resulting injuries and managements in children and adolescents between 0 and 17 years old, treated in an Emergency Room in 2019.

**Methods:**

a retrospective cross-sectional descriptive study carried out by collecting data from medical records from January to December of 2019, encompassing pediatric trauma victims, divided according to age groups: infants (0-1 year), preschool children (2-4 years), school children (5-10 years) and adolescents (11-17 years).

**Results:**

3,741 patients records were included in the study. The search for assistance occurred spontaneously in about 70% of the cases and males were the most affected at all ages. In infants and preschoolers, the main mechanism of trauma was fall from heights, corresponding to 57.2% and 34.1%, respectively, whereas in school children and adolescents, the main mechanism was ground-level falls (38%) and sports trauma (22,3%), in this order. The main injuries presented, in general, were traumatic brain injury (28,2%), upper limb contusion (23,2%) and upper limb fractures (16,3%).

**Conclusions:**

the profile of the victims analyzed indicates the male sex as the most affected, with the trauma mechanism being the differential according to age. The most frequent mechanism is falls, more prevalent in infants and preschoolers, and the most common injury is extremity contusion, with the upper limbs being the most affected. In general, the cases were considered of low complexity, with a hospitalization rate of 6%.

## RESUMO

**Objetivo:**

analisar a prevalência dos tipos de trauma, lesões decorrentes e condutas tomadas em crianças e adolescentes entre 0 e 17 anos, atendidos em um Pronto-Socorro no ano de 2019.

**Métodos:**

estudo transversal retrospectivo descritivo realizado através do levantamento de dados dos prontuários médicos no período de janeiro a dezembro de 2019, englobando pacientes pediátricos vítimas de trauma, divididos conforme a faixa etária: lactentes (0-1 ano), pré-escolares (2-4 anos), escolares (5-10 anos) e adolescentes (11-17 anos).

**Resultados:**

foram incluídos no estudo 3741 prontuários de pacientes. A procura de assistência médica ocorreu de maneira espontânea em cerca de 70% dos casos, sendo o sexo masculino o mais acometido significativamente em todas as idades. Em lactentes e pré-escolares, o principal mecanismo de trauma foi a queda de outro nível, correspondendo a 57,2% e a 34,1% das ocorrências, respectivamente; já nos escolares e adolescentes o principal mecanismo foi a queda de mesmo nível (38%) e o trauma esportivo (22,3%), nessa ordem. As principais lesões apresentadas, de maneira geral, foram o traumatismo cranioencefálico (28,2%), contusão de membros superiores (23,2%) e fratura de membros superiores (16,3%).

**Conclusões:**

o perfil das vítimas analisadas aponta o sexo masculino como o mais acometido, sendo o mecanismo de trauma o diferencial conforme a idade. Os mecanismos mais frequentes são as quedas, mais prevalentes em lactentes e pré-escolares, e a lesão mais comum é a contusão de extremidades, sendo os membros superiores os mais acometidos. Em geral, os casos foram considerados de baixa complexidade, com uma taxa de internação de 6%.

## INTRODUCTION

Trauma is currently the main cause of death in the first four decades of life^1^. By affecting mainly children and adolescents, it causes physical, psychological, and social damage that negatively interferes with the development of this population, in addition to causing feelings of guilt and loss in family members, burdening the public health system, and segregating this child or adolescent before society^2^ ^-^ ^4^.

The World Health Organization (WHO) estimates that around 950,000 deaths occur each year among children and adolescents under the age of 18, most of which are the result of preventable and unintentional events, such as traffic accidents, drowning, burns, and falls. Physical and sexual violence and neglect account for more than 200,000 deaths per year in this age group^5^. 

In Brazil, external causes of mortality and morbidity, classified according to the International Statistical Classification of Diseases and Related Health Problems, Tenth Revision (ICD-10), were responsible for 218,786 hospitalizations and 16,310 deaths of children and adolescents (zero to 19 years-old) in 2019^6^ ^,^ ^7^.

According to Chammas et al., the most common non-fatal accidents between zero and 12 years of age are, in order of prevalence, are fall from a higher level, fall from the same level, collision with an object, torsion/ physical exertion, and trauma from sharp instruments, most often occurring at home. Such information is consistent with results presented in the literature^2^ ^,^ ^8^ ^,^ ^9^.

The prevalence of types of accidents in childhood is associated with variables such as sex, age group, and neuropsychomotor development (physical and mental immaturity, inexperience, inability to foresee dangerous situations, lack of motor coordination, and body awareness)^5^ ^,^ ^10^ ^-^ ^15^. At that age, traumatic injuries are responsible for permanent disabilities, capable of altering social relationships and quality of life^3^ ^,^ ^5^ ^,^ ^16^. In addition, the economic consequences of accidents are highly relevant, due to the high cost of recovery and health care. In 2019, according to the Department of Informatics of the Brazilian Public Unified Health System (DATASUS), the total amount spent on hospitalizations due to external causes in children and adolescents alone was BRL 1,202,779.19^2^ ^,^ ^5^ ^,^ ^17^.

Most studies carried out in recent years on the Brazilian population expose isolated aspects related to pediatric mortality and morbidity, and there is a lack of sufficient data to characterize the epidemiological profile of victims and their influence on the prevalence of each type of trauma^2^ ^,^ ^8^ ^,^ ^15^ ^,^ ^18^ ^,^ ^19^.

Thus, the objective of this article is to determine the type of accident, the resulting injuries, and the actions taken, in addition to correlating the prevalence of different traumas with the profile of pediatric victims treated at a reference trauma emergency room in the city of Curitiba/ PR, in 2019.

## METHODS

We carried out this descriptive, retrospective, cross-sectional study of medical records from a reference Trauma Hospital in Curitiba-PR, from January to December 2019, encompassing pediatric (between zero and 17 years old) victims of trauma. The Hospital do Trabalhador is a general hospital, providing 24-hour care in the Emergency Room for urgencies and emergencies related to accidents and/or trauma, with access for all age groups. Clinical and surgical cases were attended by general surgeons, trauma surgeons, orthopedists, and neurosurgeons, according to sustained injuries.

The medical records were divided and analyzed according to the age group of the patients: infants (0-1 year), preschoolers (2-4 years), schoolchildren (5-10 years), and adolescents (11-17 years). The number of pediatric patients treated between January and December 2019 at the studied hospital was 15,314. Of these, we analyzed about 1,000 medical records by age group, comprising more than 4,000 medical records. When the age group had more than 1,000 patients, we randomly drew 1,000 records with Microsoft Excel, to allow the analysis of 1,000 patients in each age group.

We excluded the records of patients with clinical complaints (without trauma mechanisms, such as poisoning, airway infections, gastroenterocolitis, fever, among others), patients who evaded during care, trauma victims who returned to the hospital to perform scheduled procedures or due to complications from the clinical condition presented in a previous hospitalization (to avoid data duplication), and cases with missing or incomplete records.

The medical records were located, analyzed, and those with inclusion criteria were separated so that the following information could be obtained: attendance number, age, age group, sex, month of admission, time of day, classification according to the Manchester protocol, type of transport to the hospital, prehospital immobilization, trauma site, trauma mechanism, type of injury, complementary exams, conduct, hospitalization, and death. The entry time was divided into morning (06:00-12:00), afternoon (12:01-18:00), night (18:01-00:00), and early morning (00:00-05:59). The location of trauma was classified into home, school/day care center, and external areas (including the street, traffic, and other environments other than home, school, and day care center).

The trauma mechanisms analyzed were self-inflicted/suicide attempt, bodily assault, sexual assault, run over, electric shock, collision with an object, traffic accidents, penetration or ingestion of a foreign body, impalement, physical effort/torsion, crushing, strangulation by object, explosion of fireworks, stab wound, gunshot wound, cutting/piercing instrument, bite, straddle injury, fall from walker, fall from the same level (FSL), fall from a higher level (FHL), skateboard fall, bicycle fall, burn, upper limb traction, sports trauma, and not reported.

We collected the data in Google Forms and formatted them in graphs and tables in Microsoft Excel (2013) for further analysis, to relate the obtained variables of interest. Statistical analysis was performed with the chi square test and the p value in the sex distribution according to age group (Table 1), using the GraphPad Prism 9.5.1 program, p-values <0.05 being considered significant.

**Table 1:** Sex distribution according to age group, in absolute number (n) and percentage (%), with chi-square value (X ^2^ ) and p.

	Male		Female	
Age	n	%	n	%
Infants (0-1 years old)	490	55.7	389	44.3
Preschoolers (2-4 years old)	530	55	435	45
Schoolchildren (5-10 years old)	550	55.4	442	44.6
Teenagers (11-17 years old)	600	66.2	305	33.8
Total	2170	100	1571	100
X^2^ = 33.83, p<0.0001				

We collected all data at the Hospital’s Study Center, with access to the medical records being exclusive to the researchers participating in the study and who previously committed themselves to the confidentiality of the data by signing the Term of Commitment for Data Use. The research project was approved by the Ethics and Research Committee on August 28, 2020, under protocol number CAAE 36478020.5.0000.5225, opinion number 4.244.647.

## RESULTS

In 2019, 15,314 children between zero and 17 years of age were treated at the Hospital’s Emergency Room. We analyzed 3,741 of these records, representing a sample with a margin of error of 1.39 percentage points, with a confidence interval of 95%. We divided data collection and analysis by age group: infants (0-1 year), preschoolers (2-4 years), schoolchildren (5-10 years), and adolescents (11-17 years). Of the analyzed patients, 2,170 were male, representing 58% of the total sample.

We excluded 785 records during collection, representing 17.3% of the total number of records drawn. The main cause of exclusion was consultations due to clinical complaints, representing 45.8% of the medical excluded records, followed by evasion (20.2%), complications of a previous condition (15.6 %), and missing or incomplete information (14.2%).

**Figure 1:**
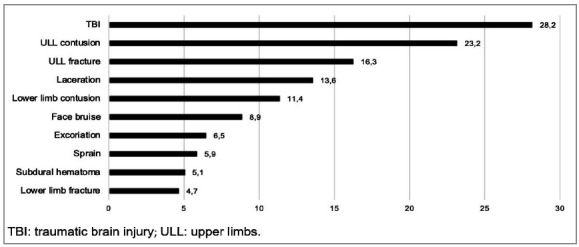
Main injuries in all age groups, described in percentage (%).

The sex distribution according to age group and the main lesions found in the analyzed population are shown, respectively, in Table 1 and in Figure 1. Information regarding hospitalizations according to the groups studied can be seen in Table 2. In the following, each age group was described separately.

**Table 2:** Hospitalization as conduct after trauma, according to age group: absolute number (n) and percentage (%) of hospitalizations, specialty responsible for admission, and need for surgery during hospitalization.

		Infants (n=879)		Preschoolers (n=965)		Schoolchildren (n=992)		Adolescents (n=905)	
		n	%	n	%	n	%	n	%
Admission		48	5,4	55	5,6	66	6,6	75	8,2
Specialty	General surgery	15	31,2	17	30,9	5	7,5	12	16
	Neurosurgery	22	45,8	10	18,1	7	10,6	3	4
	Orthopedics	8	16,6	27	49	53	80,3	60	80
	Pediatrics	3	6.2	1	1.8	1	1.5	0	0
Surgery		14	29,1	23	41,8	45	68,1	65	86,6

The statistical analysis of Table 1 found that, in all age groups, the occurrence of accidents in males is more significant statistically compared with females, confirmed by a Chi-square (X^2^) of 33.83 and p<0.0001.

### Infants

We included 879 medical records of infants. Of these, 55.7% were male (n=490) and the main entry period was during the afternoon (40.1%), followed by night (39.5%), morning (11.4%), and early morning (8.7%). Regarding the Manchester Protocol classification, 70.7% of the cases were urgent, followed by very urgent (17.1%), less urgent (10.6%), emergency (0.04%), and non-urgent (0 .02%). The classification was not informed in six records.

Most transportation means to the hospital was spontaneous (77.2%), followed by the Mobile Emergency Care Service (SAMU) (16.3%), the Integrated Emergency Trauma Care Service (SIATE) (3,4%), and white ambulance (1.7%). Transport was not reported in 11 medical records.

The trauma site was not informed in 44% of the medical records. In the 485 cases with such information present, the home environment was the most common place of trauma (84.1%), followed by external areas (14.4%) and school/day care (1.4%). The main trauma mechanism was FHL (57.2%), followed by FSL (19.2%), impact against an object (7%), and upper limb traction (5%), not being reported in 16 patients. Among infants who had accidents at home, 87.25% had falls (adding FHL, FSL and falling from a walker).

The most common injuries were Traumatic Brain Injury (TBI) (54.8%), facial contusion (18.8%), upper limb contusion (18.3%), laceration (12.5%), and excoriation (10.5%), not being reported in 23 patients. Furthermore, falls accounted for 77.3% of trauma mechanisms. Among infants who suffered falls, 64.4% had TBI. Table 3 shows sex, trauma mechanisms, and the most prevalent injuries in infants.

**Table 3:** Infants: sex, trauma mechanisms, and most prevalent injuries, in absolute number (n) and percentage (%).

Infants (n=879)		
Sex	n	(%)
Male	490	55.7
Female	389	44.3
Trauma mechanisms	n	(%)
FHL	503	57.2
FSL	169	19.2
Collision against object	62	7
Upper limb traction	44	5
Crushing	24	2.7
Injuries	n	(%)
TBI	482	54.8
Face bruise	166	18.8
ULL contusion	161	18.3
Laceration	110	12.5
Excoriation	93	10.5

FSL: fall from the same level; FHL: fall from higher level; TBI: traumatic brain injury; ULL: upper limbs.

Regarding complementary exams, X-rays were performed in 46.1% of the infants, Computed Tomography (CT) in 24.2%, Focused Assessment with Sonography for Trauma (FAST) in 2.5%, Ultrasonography (US) in 0.5%, and laboratory tests in 0.2%. No examination was performed in 30.1% of cases and the information was not reported in 11 patients. Of the infants who suffered TBI, CT was performed in 39.4% of cases. Of the patients who underwent CT, 0.04% sustained a skull fracture.

Regarding the action taken, 43.2% received only discharge with instructions, 35.9% analgesia, 11.6% remained under observation, 11% received plaster or plaster splint, 8% reduction, 6.1% suture, 5.1% received antibiotics, 2.1% received wound cleaning or dressing, 1.9% were immobilized, 1.5% went to surgery, and 0.2% of patients were referred to other hospitals. Immobilization refers to other methods other than plaster or plaster cast, such as sling, Canadian sling, metallic splint, among others.

A total of 48 infants were admitted, representing 5.4% of these patients. Most were hospitalized by the specialty of Neurosurgery, followed by General Surgery, Orthopedics, and pediatrics. Falls were the trauma mechanism in 60.4% of hospitalizations. The length of hospital stay ranged from one to 20 days, with 68.7% of the infants remaining hospitalized for one or two days. One death was reported.

### Preschoolers

We analyzed 965 records of preschoolers. Of these, 55% were male (n=530). Most were admitted at night (42.7%), followed by afternoon (37.3%), morning (12.4%), and early morning (7.4%). Most patients were classified as urgent (71.2%), 16.6% as very urgent, 10% as less urgent, 0.6% as emergency. and 0.1% as non-urgent. The classification was not informed in five patients.

Regarding transportation, 78.9% of the patients came spontaneously, 13% via SAMU, 4.1% via SIATE, 3.7% via white ambulance, the transport not being reported in just one case.

The location of the accidents was not informed in 26.6% of the cases. In the 708 records in which the location was identified, most accidents occurred at home (53.1%), followed by external areas (39.1%) and school/nursery (7.7%). The most common trauma mechanism was FHL (34.1%), followed by FSL (29.7%) and collision with an object (11.6%). Such information was not reported in three medical records. Falls were the main mechanism of trauma in the home environment, representing 75.7% of the causes of trauma in that place.

TBI was the most common trauma (29.5%), followed by upper limb contusion (25.9%), laceration (19.7%), and upper limb fracture (15.5%). No injuries were reported in 27 medical records. In all settings, falls accounted for 63.9% of trauma mechanisms. Among preschoolers who suffered falls, 36.5% suffered TBI. Moreover, about 86.6% of upper limb fractures and 79.3% of clavicle fractures had falls as the causative mechanism. Sex, trauma mechanisms, and injuries most found in preschoolers can be seen in Table 4.

**Table 4:** Preschoolers: sex, trauma mechanisms, and most prevalent injuries, in absolute number (n) and percentage (%).

Preschoolers (n=965)		
Sex	n	(%)
Male	530	55%
Female	435	45%
Trauma mechanisms	n	(%)
FHL	330	34.1
FSL	287	29.7
Collision against object	112	11.6
Crushing	45	4.6
Physical effort / Twisting	37	3.8
Injuries	n	(%)
TBI	285	29.5
ULL contusion	250	25.9
Laceration	191	19.7
ULL fracture	150	15.5
Subdural hematoma	86	8.9

FSL: fall from the same level; FHL: fall from higher level; TBI: traumatic brain injury; ULL: upper limbs.

Regarding exams, X-rays were performed in 62% of the cases, CT in 17.6%, FAST in 4.4%, laboratory tests in 0.7%, and US in 0.2%. No examination was performed in 22.6% of the patients. CT was performed in 50.8% of patients who suffered TBI. Of those undergoing CT, 0.01% suffered a skull fracture.

As for the conduct, 37% received plaster or plaster cast, 16.9% analgesia, 13.3% suture, 7% reduction, 6.8% immobilization, 5.5% cleaning and dressing, 3.3% remained under observation, 2.3% went to surgery, 0.8% received antibiotic therapy, and 0.5% were referred to other hospitals. Discharge with instructions happened in 0.5% of cases.

Fifty-five patients were hospitalized, totaling 5.6% of preschoolers. Of these, most were admitted for Orthopedics, followed by General Surgery, Neurosurgery, and pediatrics. Of the hospitalized patients, 47.2% had falls as the causative mechanism. The number of days of hospitalization varied between one and seven, with one day of hospitalization representing 80.7% of the cases. There were two reported deaths.

### Schoolchildren

We analyzed 992 records of school patients. Of these, 55.4% were male (n=550). The night period was the time with the most admissions (42.8%), followed by the afternoon (39.2%), morning (13.4%), and early morning (4.5%). Most consultations were classified as urgent (57%), 27.9% as less urgent, 14.4% as very urgent, and 0.1% as emergency. The classification was absent in five records.

About 81.4% of the students came to the emergency room spontaneously, 8.6% by SAMU, 5.8% by SIATE, 3.6% by white ambulance, not being reported in four medical records.

The trauma site was not informed in 69.5% of the medical records. Of the 302 patients with location information, external areas were the most common site of trauma (58.6%), followed by school (21.8%), and home (19.5%). The main trauma mechanism was FSL (38%), followed by FHL (15.1%), impact against an object (13.5%), physical exertion/twisting (8.8%), bicycle fall (6.4%), and sports trauma (6%). The mechanism was not reported in three records.

The most common injury was upper limb fracture (25.7%), followed by TBI (18.8%), upper limb contusion (18.6%), lower limb contusion (11.9%), laceration (11%), and sprains (8.3%). Falls accounted for 53.1% of trauma mechanisms and, among all students who suffered falls, the most common injury was upper limb contusion (54.8%). Of the students who suffered trauma to the upper limb, 64% had an upper limb fracture and, among these, 16% underwent surgery.

The main mechanisms of trauma in the school environment were falls (56%), followed by collisions with objects (25.7%). In descending order, the most common injuries in this environment were upper limb contusion, TBI, facial contusion, and upper limb fracture. In addition, about 64.1% of TBI cases had falls as the causative mechanism. Table 5 shows sex, trauma mechanisms, and the most common injuries observed in schoolchildren.

**Table 5:** Schoolchildren: sex, trauma mechanisms, and most prevalent injuries, in absolute number (n) and percentage (%).

Students (n=992)		
Sex	n	(%)
Male	550	55.4
Female	442	44.6
Trauma mechanisms	n	(%)
FSL	377	38
FHL	151	15.1
Collision against object	134	13.5
Physical Effort/Twist	88	8.8
Bike fall	64	6.4
Injuries	n	(%)
ULL fracture	255	25.7
TBI	187	18.8
ULL contusion	185	18.6
Lower limb contusion	119	11.9
Laceration	110	11

FSL: fall from the same level; FHL: fall from higher level; TBI: traumatic brain injury; ULL: upper limbs.

Regarding the tests requested, 82.3% of the schoolchildren underwent X-rays, 12.3% CT, 3.9% FAST, 0.8% laboratory tests, and 0.5% US. Exams were not performed in 9.2% of cases. As for the approach taken, most patients received analgesia (83.9%), 28.5% received plaster or plaster cast, 7.7% immobilization, 7% suture, 6% antibiotic therapy, 4.6% remained under observation, 4.5% underwent surgery, 1.8% underwent cleaning and dressing, and 1.6% underwent reduction. Among all, 8.3% received only discharge with guidance as conduct.

A total of 66 schoolchildren were hospitalized, accounting for 6.6% of the patients. Of these, most were admitted for Orthopedics, followed by Neurosurgery, General Surgery, and pediatrics. The length of stay varied between one and 16 days, with 46.9% being hospitalized for only one day and 28.7% for two days. There was one reported death. Among hospitalized patients, 63.6% had falls as a trauma mechanism.

### Teenagers

We analyzed 905 medical records of adolescents, males being the most prevalent, representing 66.2% of cases (n=600). The afternoon period was the time with the most admissions (38.3%), followed by night (37.9%), morning (16.7%), and early morning (6.9%). Most patients were classified as less urgent (50.3%), followed by urgent (35.6%), very urgent (13.1%), emergencies (0.5%), and non-urgent (0. 1%). One medical record lacked the classification.

About 80% of the patients came to the emergency room spontaneously, 9.2% by SAMU, 8.3% by SIATE, and 2.2% by white ambulance. The trauma site was not informed in 47.5% of the medical records. Of the 475 patients with the accident site described, external areas were the most common (80.6%), followed by home (13.6%), and school environments (5.6%). The main trauma mechanism was sports trauma (22.3%), followed by FSL (20.5%), physical exertion/torsion (11.2%), impact against an object (10.2%) and FHL (7.4%). Such information was not described in two medical records.

The most common injury was upper limb contusion (30%), followed by lower limb contusion (19.4%), upper limb fracture (17.3%), TBI (12.4%), sprain (12 .2%), laceration (10.8%), and lower limb fracture (7.8%). In six records, no injuries were described. The most common mechanism in external areas was sports trauma, followed by traffic accidents, bicycle falls, and falls. Sex, trauma mechanisms, and the most prevalent injuries in adolescents are described in Table 6.

**Table 6:** Adolescents: sex, trauma mechanisms and most prevalent injuries, in absolute number (n) and percentage (%).

Teenagers (n=905)		
Sex	n	(%)
Male	600	66.2
Female	305	33.8
Trauma mechanisms	n	(%)
Sports trauma	202	22.3
FSL	186	20.5
Physical effort / Twisting	102	11.2
Collision against object	93	10.2
FHL	67	7.4
Injuries	n	(%)
ULL contusion	272	30
Lower limb contusion	176	19.4
ULL fracture	157	17.3
TBI	113	12.4
Sprain	111	12.2

FSL: fall from the same level; FHL: fall from higher level; TBI: traumatic brain injury; ULL: upper limbs.

Falls accounted for 27.9% of all trauma mechanisms. Among all adolescents who suffered falls, the most common injury was trauma to the upper limb (53.3%). Of those who suffered an upper limb contusion, 48.1% had an upper limb fracture. Regarding sports trauma, upper limb fracture was present in 41.8% of patients. Of these, 6.5% required surgery to correct the fracture.

The most requested test was radiography, performed in 87.9% of patients, followed by CT (12%), FAST (5.7%), laboratory tests (1.3%), and US (0.1%). Exams were not performed in 5.5% of cases. Regarding the conduct, 66.4% of the patients received analgesia, 22.7% plaster or plaster splint, 12% immobilization, 7.1% underwent surgery, 3.7% underwent reduction, 2% received cleaning and dressing, and 2 % remained under observation. Only 9.3% of the adolescents were discharged with guidance and 0.1% were referred to other hospitals.

Seventy-five teens were hospitalized, totaling 8.2% of the patients. Of these, most were admitted for Orthopedics, followed by General Surgery, and Neurosurgery. The length of stay varied between one and 36 days, with 62.6% of the patients being hospitalized for one and two days. Among hospitalized patients, 28% had falls as a trauma mechanism. There were three reported deaths.

## DISCUSSION

The results of the present study demonstrated a higher prevalence of trauma in male patients, corresponding to 58% of the medical records included. This data is similar to that found in the study by Luiz et al., in which 56.4% were male^20^. Other studies^9^ ^,^ ^21^ found similar figures. The higher prevalence of accidents among boys is probably due to the difference between the sexes regarding behavior, education, and the types of games they play^5^ ^,^ ^10^ ^-^ ^15^ ^,^ ^20^ ^,^ ^22^. 

We could not evaluate which age group is most affected, since we obtained similar samples from the four age groups. However, in a global analysis, the main trauma mechanism found were falls (FSL, FHL and falling from a walker), corresponding to 55.5% of the total of 3,741 patients analyzed. According to other studies in the literature^8^ ^,^ ^9^ ^,^ ^20^, falls are the most prevalent type of injury in the pediatric population, corresponding to 60.2% and 56.6% of traumas, respectively. When evaluating this information by age group, it became clear that falls were the most prevalent trauma mechanism among infants (77.3%) and preschoolers (63.8%), which can be explained by their neuropsychomotor development and the inability of infants to distinguish whether their actions may pose a risk to their physical integrity^10^ ^-^ ^13^. This reinforces the need for greater surveillance of these children, especially in the home environment, where falls were responsible for 87.25% of accidents in the infant population^8^ ^,^ ^9^ ^,^ ^15^ ^,^ ^18^ ^,^ ^19^.

We observed that the second main trauma mechanism among all infants was collision with objects, corresponding to approximately 11% of the total. This differs from other studies^20^ ^,^ ^22^, in which exogenous intoxications and foreign body penetration took second place. In the present study, foreign body penetration was considered insignificant, with a total prevalence of less than 0.5%. Exogenous intoxications were considered clinical complaints and, therefore, were not included. This may explain the differences in the prevalence of these types of accidents in other articles, which included childhood accidents and not just trauma.

Sports trauma was the third most common mechanism, representing approximately 7.2% of the total sample. Its prevalence was higher in schoolchildren and especially in teenagers, an age group in which sports practice becomes more frequent, which may involve activities of greater physical contact or risk of falling.

The main injuries identified in the total sample corroborate the main trauma mechanisms found. Extremity contusion was the most common injury among the pediatric population, present in 47.8% of cases. The upper limbs were the most injured, approximately 2.2 times more than the lower limbs. The second most prevalent injury was TBI (28.5%), in which infants were the most affected. Fractures of the extremities were the third most common injury, the upper limbs being 4.4 times more affected than the lower ones.

The results obtained reveal that most cases were of low complexity, with a hospitalization rate of approximately 6%. Therefore, most patients were treated and discharged on the same day, requiring only outpatient procedures (bandage, suture, plaster/splint, and immobilization) or analgesia and support measures. Andrade et al. emphasize early hospital discharge in 94% of the patients treated^22^.

In the present study, 62.6% of hospitalizations were performed for surgical procedures. Of these, 81.8% were performed by the Orthopedics team and 13.6% by the hospital’s General Surgery team. Furthermore, Orthopedics was the specialty with the highest rate of hospitalizations, accounting for 60.1% of all admitted children, followed by General Surgery (20.7%) and Neurosurgery (17%). This higher prevalence is justified by the high rate of injuries involving extremities. 

The lack of information in the analyzed medical records hampered the analysis of the data pertaining to the trauma site. However, based on the results obtained (n=1,970), there is an equivalent prevalence between accidents occurring in the home environment and in external areas (both with 46%). We observed a higher prevalence of home accidents in younger populations (infants and preschoolers). Such information strengthens the need to guide caregivers regarding the safety of infants at home. In the external environment, traffic accidents were responsible for a quarter of the trauma mechanism. In a study conducted in Sergipe, 60.3% of accidents occurred on public roads^9^. This data reinforces the importance of preventing traffic accidents, either by guiding the use of qualified safety equipment, or through campaigns to prevent car accidents on a national scale.

## CONCLUSION

Most accidents in the 0-17 age group involve males, with the trauma mechanism varying according to age. Falls correspond to 55.5% of the analyzed mechanisms and mainly affect infants and preschoolers in the home environment. The second most prevalent mechanism, unlike what is reported in the literature, was collision with objects. Foreign body penetration was considered insignificant and exogenous intoxications were considered clinical complaints, which may be an explanation for this disagreement with other articles. In older children, schoolchildren and adolescents, sports trauma was the most frequent. Upper limb contusion was the most common injury among all ages, followed by TBI, in which infants were the most affected. In younger children (infants and preschoolers), most traumas occurred at home, and a similar incidence was reported between home and outdoors among schoolchildren and adolescents. Traffic accidents were responsible for a quarter of traumas in external areas. Most cases were considered of low complexity and reflected a small hospitalization rate (6%).
